# Inference of protein-protein interaction networks from multiple heterogeneous data

**DOI:** 10.1186/s13637-016-0040-2

**Published:** 2016-02-19

**Authors:** Lei Huang, Li Liao, Cathy H. Wu

**Affiliations:** 1 Department of Computer and Information Sciences, University of Delaware, 18 Amstel Avenue, Newark, 19716 DE USA; 2 Center for Bioinformatics and Computational Biology, University of Delaware, 15 Innovation Way, Newark, 19711 DE USA

**Keywords:** Protein interaction network, Network inference, Interaction prediction, Differential evolution

## Abstract

Protein-protein interaction (PPI) prediction is a central task in achieving a better understanding of cellular and intracellular processes. Because high-throughput experimental methods are both expensive and time-consuming, and are also known of suffering from the problems of incompleteness and noise, many computational methods have been developed, with varied degrees of success. However, the inference of PPI network from multiple heterogeneous data sources remains a great challenge. In this work, we developed a novel method based on approximate Bayesian computation and modified differential evolution sampling (ABC-DEP) and regularized laplacian (RL) kernel. The method enables inference of PPI networks from topological properties and multiple heterogeneous features including gene expression and Pfam domain profiles, in forms of weighted kernels. The optimal weights are obtained by ABC-DEP, and the kernel fusion built based on optimal weights serves as input to RL to infer missing or new edges in the PPI network. Detailed comparisons with control methods have been made, and the results show that the accuracy of PPI prediction measured by AUC is increased by up to 23 %, as compared to a baseline without using optimal weights. The method can provide insights into the relations between PPIs and various feature kernels and demonstrates strong capability of predicting faraway interactions that cannot be well detected by traditional RL method.

## Introduction

Uncovering protein-protein interaction (PPI) is crucial to having a better understanding of intracellular signaling pathways, modeling of protein complex structures and elucidating various biochemical processes. Although several high-throughput experimental methods, such as yeast two-hybrid system and mass spectrometry method, have been used to determine a larger number of protein interactions, these methods are known to be prone to having high false-positive rates, besides of their high cost. Therefore, efficient and accurate computational methods for PPI prediction are urgently needed.

Generally, current computational methods for PPI prediction can be classified into two categories: A) pair-wise biological similarity based methods and B) network level-based methods. For category A, computational approaches have been developed to predict if any given pair of proteins interact with each other, based on various properties such as sequence homology, gene co-expression and phylogenetic profiles [1–5]. Moreover, some previous work also demonstrated that three-dimensional structural information, when available, can be used to predict PPIs with accuracy superior to predictions based on non-structural evidence [6, 7]. However, with no first principles to tell deterministically yet if two given proteins interact or not, the pair-wise biological similarity based on various features and attributes can run out its predictive power, as often the signals may be too weak or noisy. Therefore, recently, many researches have been focused on integrating heterogeneous pair-wise features, e.g., genomic features, semantic similarities, in seek of better prediction accuracy [8–11]. It is biologically meaningful if we can disentangle the relations among various pair-wise biological similarities and PPIs, but it is still in early stage for the incomplete and noisy pair-wise similarity kernels.

To circumvent the limitations with using pair-wise biological similarity, efforts have also been made to investigate PPI prediction in the context of networks, which may provide extra information to resolve ambiguities incurred at pairwise level. A network can be constructed from reliable pair-wise PPIs, with nodes representing proteins and edges representing interactions. Topological features, such as the number of neighbors, can be collected for nodes and then are used to measure the similarity for any given node pair to make PPI prediction for the corresponding proteins [12–15]. Inspired by the PageRank algorithm [16], variants of random walk-based methods have been proposed to go beyond these node centric topological features to get the whole network involved; the probability of interaction between given two proteins is measured in terms of how likely a random walk in the network starting at one node will reach the other node [17–19]. These methods are suitable for PPI prediction in cases when the task is to find all interacting partners for a particular protein, by using it as the start node for random walks. The computational cost increases from *O*(*N*) to *O*(*N* ^2^) for all-against-all PPI prediction. To overcome the limitation of single start-node random walk, many kernels on network for link prediction and semi-supervised classification have been systemically studied [20], which can measure the random-walk distance for all node pairs at once. Compared with the random walk methods, kernel methods are obviously more efficient and applicable to various network types. But, both the variants of random walk and random walk-based kernels cannot differentiate faraway interacting candidates well. Besides, instead of computing proximity measures between nodes from the network structure directly, Kuchaiev et al. and Cannistraci et al. proposed geometric de-noise methods that embed PPI network into a low-dimensional geometric space, in which protein pairs that are closer to each other represent good candidate interactions [1, 21].

Furthermore, when the network is represented as an adjacent matrix, the prediction problem can be transformed into a spectral analysis and matrix completion problem. For example, Symeonidis et al. [22] did link prediction for biological and social networks based on multi-way spectral clustering. Wang et al. [23] and Krishna et al. [24] predicted PPI interactions through matrix factorization-based methods. By and large, the prediction task will be reduced to convex a optimization problem, and the performance depends on the objective function, which should be carefully designed to ensure fast convergence and avoidance of being stuck in the local optima.

The two kinds of methods, pair-wise biological similarity-based methods and network level-based methods, can be mutually beneficial. For example, weights can be assigned to edges in the network using pair-wise biological similarity scores. In Backstrom et al. [19], a supervised learning task is proposed to learn a function that assigns weighted strengths to edges in the network such that a random walker is more likely to visit the nodes to which new links will be created in the future. The matrix factorization-based methods proposed by Wang et al. [23] and Krishna et al. [24] also included multi-modal biological sources to enhance the prediction performance. In these methods, however, only the pair-wise features for the existing edges in the network will be utilized, even though from a PPI prediction perspective, what is particularly useful is to incorporate pair-wise features for node pairs that are not currently linked by a direct edge but will if a new edge (PPI) is predicted. Therefore, it would be of great interest if we can infer PPI network directly from multi-modal biological features kernels that involve all node pairs. In Yamanishi et al. [25], a method is developed to infer protein networks from multiple types of genomic data based on a variant of kernel canonical correlation analysis (CCA). In that work, all genomic kernels are simply added together, with no weights to regulate these heterogeneous and potentially noisy data sources for their contribution towards PPI prediction. Also, it seems that the partial network needed for supervised learning based on kernel CCA needs to be sufficiently large, e.g., a leave-one-out cross validation is used, to attain good performance.

In this paper, we propose a new method based on ABC-DEP sampling method and regularized Laplacian (RL) kernel to infer PPI networks from multiple hetergeneous data. The method uses both topological features and various genomic kernels, which are weighted to form a kernel fusion. The weights are optimized using ABC-DEP sampling [26]. Unlike data fusion with genomic kernels for binary classification [27], the combined kernel in our case will be used instead to create a regularized Laplacian kernel [20, 28] for PPI prediction. We demonstrate how the method circumvents the issue of unbalanced data faced by many machine-learning methods in bioinformatics. One main advantage of our method is that only a small partial network is needed for training in order to make the inference at the whole network level. Moreover, the results show that our method works particularly well with detecting interactions between nodes that are far apart in the network, which has been a difficult task for other methods. Tested on Yeast PPI data and compared to two control methods, traditional regularized Laplacian kernel method and regularized Laplacian kernel based on equally weighted kernels, our method shows a significant improvement of over 20 % increase in performance measured by ROC score.

## Methods and data

### Problem definition

Formally, a PPI network can be represented as a graph *G*=(*V,E*) with *V* nodes (proteins) and *E* edges (interactions). *G* is defined by the adjacency matrix *A* with *V*×*V* dimension:

(1) $$ {A_{i,j}} = \left\{ \begin{array}{c} 1, {if}\, {(i,j)}\in{E} \\ 0, {if}\, {(i,j)}\notin{E} \\ \end{array} \right.\,  $$

where *i* and *j* are two nodes in the nodes set *V*, and (*i,j*) represents an edge between *i* and *j*, (*i,j*)∈*E*. The graph is called *connected* if there is a path of edges to connect any two nodes in the graph. For supervised learning, we divide the network into three parts: connected training network *G*_*tn*_=(*V*,*E*_*tn*_), validation set *G*_*vn*_=(*V*_*vn*_,*E*_*vn*_), and testing set *G*_*tt*_=(*V*_*tt*_,*E*_*tt*_). For *G*_*tn*_, it consists of a minimum spanning tree, augmented with a small set of randomly selected edges. Because all edges are equally weighted, each time a minimum spanning tree is newly built, it will be different from a previous one. And *G*_*vn*_ and *G*_*tt*_ are two non-overlapping subsets of edges randomly chosen from the edges that are not in *G*_*tn*_.

A kernel is a symmetric positive definite matrix *K*, whose elements are defined as a real-valued function *K*(*x, y*) satisfying *K*(*x, y*)=*K*(*y, x*) for any two proteins *x* and *y* in the data set. Intuitively, the kernel for a given dataset can be regarded as a measure of similarity between protein pairs with respect to the biological properties, from which kernel function takes its value. Treated as an adjacency matrix, a kernel can also be thought of as a complete network in which all the proteins are connected by weighted edges. Kernel fusion is a way to integrate multiple kernels from different data sources by a linear combination. For our task, this combination is made of the connected training network and various feature kernels *K*_*i*_,*i*=1,2,3…*n* by optimized weights *W*_*i*_,*i*=0,1,2,3…*n*, which formally is defined by Eq. (2)

(2) $$ K_{fusion} = W_{0}G_{tn} + \sum\limits_{i=1}^{n} W_{i}K_{i}  $$

Note that the training network is incomplete, i.e., with many edges taken away and reserved as testing examples. Therefore, our inferring task is to predict or recover the interactions in the testing set *G*_*tt*_ based on the kernel fusion.

### How to infer PPI network?

**Fig. 1:**
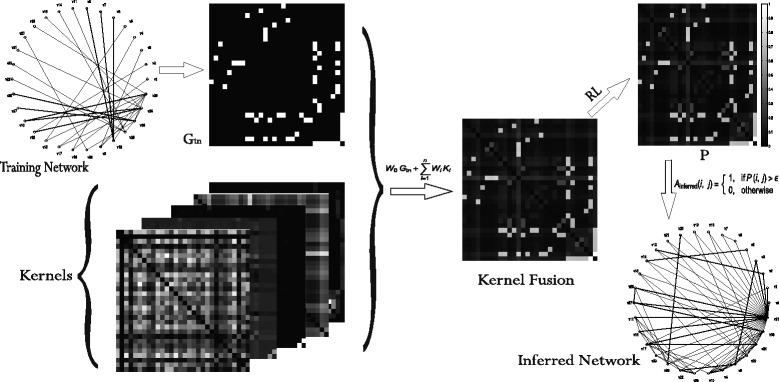
An example to show the inference process. The example comprises of a small module in the DIP yeast PPI network, which consists of protein P25358 (ELO2, elongation of fatty acids protein 2) and its 1∼3 hops away neighbors. The kernel fusion and the regularized Laplacian are shown as heatmap. The *lighter* a cell is, the more likely the corresponding proteins interact



Once the kernel fusion is obtained, it will be used to make PPI inference, in the spirit of random walk. However, instead of directly doing random walk, we apply regularized Laplacian (RL) kernel to the kernel fusion, which allows for PPI inference at the whole network level. The regularized Laplacian kernel [28, 29] is also called the normalized random walk with restart kernel in Mantrach et al. [30] because of the underlying relations to the random walk with restart model [17, 31]. Formally, it is defined as Eq. (3)

(3) $$ \textit{RL} = \sum\limits_{k=0}^{\infty} \alpha^{k}{(-L)}^{k} = {(I+\alpha\ast L)}^{-1}  $$

where *L*=*D*−*A* is the Laplacian matrix made of the adjacency matrix *A* and the degree matrix *D*; and 0<*α*<*ρ*(*L*)^−1^ where *ρ*(*L*) is the spectral radius of *L*. Here, we use kernel fusion in place of the adjacent matrix, so that various feature kernels in Eq. (2) are incorporated in influencing the random walk with restart on the weighted networks [19]. With the regularized Laplacian matrix, no random walk is actually needed to measure how “close” two nodes are and then use that closeness to infer if the two corresponding proteins interact. Rather, *RL*_*K*_ is the inferred matrix, and is interpreted as a probability matrix *P* in which *P*_*i*,*j*_ indicates the probability of an interaction for protein *i* and *j*. Algorithm 1 shows the general steps to infer PPI network from a optimal kernel fusion. Figure 1 contains a toy example to show the process of inference, where both the kernel fusion and the regularized Laplacian are shown as heatmap. The lighter a cell is, the more likely the corresponding proteins. However, to ensure good inference, it is important to learn optimal weights for *G*_*tn*_ and various *K*_*i*_ to build kernel fusion *K*_*fusion*_. Otherwise, given the multiple heterogeneous kernels from different data sources, the kernel fusion without optimized weights is likely to generate erroneous inference on PPI.

### ABC-DEP sampling method for learning weights

In this work, we revise the ABC-DEP sampling method [26] to optimize the weights for kernels in Eq. (2). ABC-DEP sampling method, based on approximate Bayesian computation with differential evolution and propagation, shows strong capability of accurately estimating parameters for multiple models at one time. The parameter optimization task here is relatively easier than that in [26] as there is only one RL-based prediction model. Specifically, given the connected training network *G*_*tn*_ and *N* feature kernels in Eq. (2), the length of the particle in ABC-DEP would be *N*+1, where particle can also be seen as a sample including the *N*+1 weight values. As mentioned before, the PPI network is divided into three parts: the connected training network *G*_*tn*_, validation set *G*_*vn*_ and testing set *G*_*tt*_. To obtain the optimal particle(s), a population of particles with size *N*_*p*_ is intialized, and ABC-DEP sampling is run iteratively until a particle is found in the evolving population that maximizes the AUC of inferring training network *G*_*tn*_, validation set *G*_*vn*_. The validation set *G*_*vn*_ is used to avoid over-fitting as the algorithm converges. Algorithm 2 shows the detailed sampling process.



Algorithm 2 is the main structure in which a population of particles with equal importance is initialized and each particle consists of kernel weights randomly generated from a uniform prior. Given the particle population, Algorithm 3 samples through the parameter space for good particles and assigns them weights according to the predicting quality of their corresponding kernel fusion *K*_*fusion*_. Note that, different from the ABC-DEP sampling method in [26] where the logarithm of the Boltzmann distribution is adopted, here, we accept or reject a new candidate particle based on Boltzmann distribution with simulated annealing method [32]. Through the evolution process, bad particles will be filtered out and good particles will be kept for the next generation. We repeat this process until the algorithm converges. The optimal particle is used to build kernel fusion *K*_*fusion*_ for PPI prediction.



### Data and kernels

We use yeast PPI networks downloaded from DIP database (Release 20150101) [33] to test our algorithm. Notably, some interactions without Uniprotkb ID have been filtered out in order to do name mapping and make use of genomic similarity kernels [27]. As a result, the PPI network contains 5093 proteins and 22,423 interactions, from which the largest connected component is used to serve as golden standard network. It consists of 5030 proteins and 22,394 interactions. Only tens of proteins and interactions are not included in the largest connected component, which makes the golden standard data almost as complete as the original network. As mentioned before, the golden standard PPI network is divided into three parts that are connected training network *G*_*tn*_, validation set *G*_*vn*_ and testing set *G*_*tt*_, where training network *G*_*tn*_ is included in the kernel fusion, validation set *G*_*vn*_ is used to find optimal weights for feature kernels and testing set *G*_*tt*_ is used to evaluate the inference capability of our method.

Six feature kernels are obtained from http://noble.gs.washington.edu/proj/sdp-svm/ for this study and the following list is about the detailed information of these kernels.

- *G*_*tn*_: *G*_*tn*_ is the connected training network that provides connectivity information. It can also be thought of as a base network to do the inference.
- *K*_*Jaccard*_ [34]: This kernel measure the similarity of protein pairs *i*,*j* in term of $\frac {neigbors(i) \cap neighbors(j)}{neighbors(i) \cup neighbors(j)}$.
- *K*_*SN*_: It measures the total number of neighbors of protein *i* and *j*, *K*_*SN*_=*neighbors*(*i*)+*neighbors*(*j*).
- *K*_*B*_ [27]: It is a sequence-based kernel matrix that is generated using the BLAST [35].
- *K*_*E*_ [27]: This is a gene co-expression kernel matrix constructed entirely from microarray gene expression measurements.
- *K*_*Pfam*_ [27]: This is a generalization of the previous pairwise comparison-based matrices in which the pairwise comparison scores are replaced by expectation values derived from hidden Markov models (HMMs) in the Pfam database [36].

These kernels are positive semi-definite. Please refer to [27] for detailed analysis (or proof). Moreover, Eq. (2) is guaranteed to be positive semi-definite, because basic algebraic operations such as addition, multiplication, and exponentiation preserve the key property of positive semi-definiteness [37]. Finally, all these kernels are normalized to the scale of (0,1) in order to avoid bias.

## Results and discussion

### Inferring PPI network

**Fig. 2:**
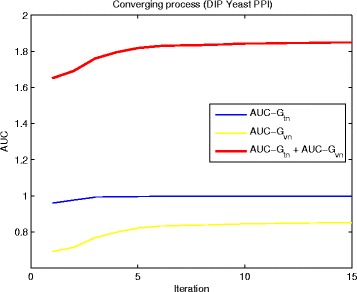
The converging process of ABC-DEP sampling used to obtain optimal weights

To show how well our method can infer PPI network from the kernel fusion, we make the task challenging by dividing the golden standard yeast PPI network into the following three parts: the connected training network *G*_*tn*_ has 5030 nodes and 5394 edges, the validation set *G*_*vn*_ has 1000 edges, and the testing set *G*_*tt*_ has 16,000 edges. This means that we need to infer and recover a large number of testing edges based on the kernel fusion and a small validation set. Firstly, we check the converging process of finding the optimal weights that used to combine feature kernels, which is shown by the Fig. 2. It clearly shows that when the AUC of predicting the training network *G*_*tn*_ reaches to 1 quickly, but the AUC of predicting the validation set *G*_*vn*_ is still in an upward trend. So *G*_*tn*_ alone cannot guarantee the optimality of the weights when the algorithm converges, which is the reason the validation set *G*_*vn*_ is used. After several iterations, the ABC-DEP algorithm is converged when both AUCs have become steady.

**Fig. 3:**
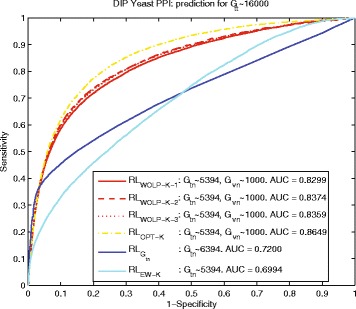
ROC curves of predicting *G*_*tt*_∼16,000 by $RL_{G_{\textit {tn}}\sim 5394}$, *RL*_*OPT-K*_, *RL*_*EW-K*_, and *RL*_*WOLP-K-i*_

With the optimal weights obtained from ABC-DEP sampling, we build the kernel fusion *K*_*fusion*_ by Eq. (2). PPI network inference is made with RL kernel Eq. (3). The performance of inference is evaluated by how well the testing set *G*_*tt*_ is recovered. Specifically, all node pairs are ranked in decreasing order by their edge weights in the RL matrix, and edges in the testing set *G*_*tt*_ are then labeled as positive and node pairs with no edges in *G* are labeled as negative. A ROC curve is plotted for true positive vs. false positives, by running down the ranked list of node pairs. Figure 3 shows the ROC curves and AUCs for three PPI network inferences: *RL*_*OPT-K*_, $ RL_{G_{\textit {tn}}} $, and *RL*_*EW-K*_, where *RL*_*OPT-K*_ indicates the RL-based PPI inference is from kernel fusion that built by optimal weights, $ RL_{G_{\textit {tn}}} $ indicates RL-based PPI inference is solely from the training network *G*_*tn*_, and *RL*_*EW-K*_ represents RL-based PPI inference is from kernel fusion built by equal weights, e.g., *W*_*i*_=1,*i*=0,1…*n*. Additionally, *G*_*set*_∼*n* indicates that there is *n* number of edges in the set *G*_*set*_, e.g., *G*_*tn*_∼5394 means the connected training network *G*_*tn*_ contains 5394 edges. As shown by Fig. 3, the PPI reference *RL*_*OPT-K*_ based on our method significantly outperforms the other two control methods, with a 20 % increase over $ RL_{G_{\textit {tn}}} $ and a 23.6 % over *RL*_*EW-K*_ in terms of AUC. It is noted that the AUC of PPI inference *RL*_*EW-K*_ based on the equally weighted built kernel fusion is even worse than that of $ RL_{G_{\textit {tn}}} $ based on a really small training network. It means there should be a lot of noises if we just naively combine different feature kernels to do PPI prediction. Our method provides an effective way to make good uses of various features for improving PPI prediction performance.

In Fig. 3, we also compared with another method, WOLP, which uses linear programming to optimize the weights *W*_*i*_ for the various kernel features [38]. It can be seen that WOLP, with AUC at about 0.83, also performs signigicantly better than the baseline, indicating that the method is effective in weighting various features to improve PPI inference. Note that although reference [38] has “random walk” in its title, the method WOLP does not do sampling; instead, the weights for kernel features are optimized by linear programming, constrained with the transition matrix from the training network for any would-be random walk over the PPI network when kernel features are incorporated. As such, WOLP is more computationally efficient but with a trade-off of slightly worse performance as compared to ABC-DEP, which has the best AUC, 0.86, in this study.

### Effects of the training data

**Fig. 4:**
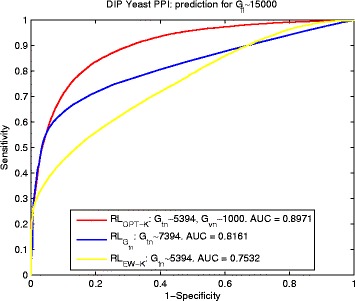
ROC curves of predicting *G*_*tt*_∼15,000 by $ RL_{G_{\textit {tn}}\sim 7394} $, *RL*_*OPT-K*_, and *RL*_*EW-K*_

**Fig. 5:**
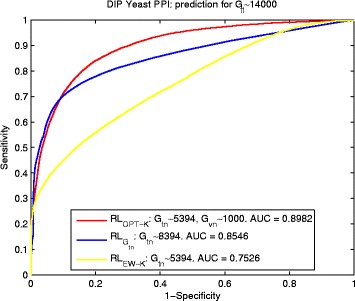
ROC curves of predicting *G*_*tt*_∼14,000 by $ RL_{G_{\textit {tn}}\sim 8394} $, *RL*_*OPT-K*_, and *RL*_*EW-K*_

**Fig. 6:**
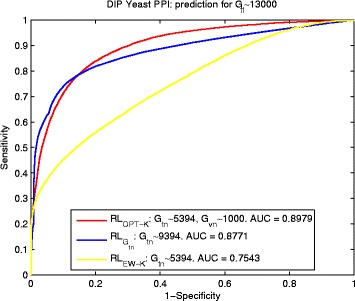
ROC curves of predicting *G*_*tt*_∼13,000 by $ RL_{G_{\textit {tn}}\sim 9394} $, *RL*_*OPT-K*_, and *RL*_*EW-K*_

**Fig. 7:**
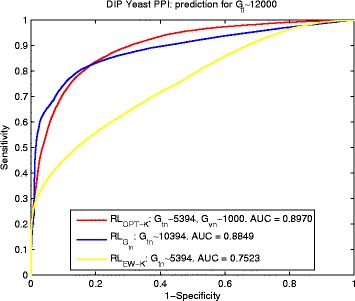
ROC curves of predicting *G*_*tt*_∼12,000 by $ RL_{G_{\textit {tn}}\sim 10394} $, *RL*_*OPT-K*_, and *RL*_*EW-K*_

Usually, given a golden standard data, we need to retrain the prediction model for different divisions of training sets and testing sets. However, if optimal weights have been found for building kernel fusion, our PPI network inference method enable us to train the model once, and do prediction or inference for different testing sets. To demonstrate that, we keep the two PPI inferences *RL*_*OPT-K*_ and *RL*_*EW-K*_ obtained before (in last section) unchanged and evaluate the prediction ability for different testing sets. We also examine how performance is affected by sizes of various sets. Specifically, while the size of training network *G*_*tn*_ for $ RL_{G_{\textit {tn}}} $ increases, sizes of *RL*_*OPT-K*_ and *RL*_*EW-K*_ are kept unchanged. Therefore, we design several experiments by dividing the golden standard network into $ G_{\textit {tn}}^{i} $ and $ G_{\textit {tt}}^{i} $, *i*=1,…,*n*, and building PPI inference $ RL_{G_{\textit {tn}}^{i}} $ to predict $ G_{\textit {tt}}^{i} $ for every time. To make comparison, we also use *RL*_*OPT-K*_ and *RL*_*EW-K*_ to predict $ G_{\textit {tt}}^{i} $. Figure 4 shows the ROC curves of predicting *G*_*tt*_∼15000 by $ RL_{G_{\textit {tn}}\sim 7394} $, *RL*_*OPT-K*_ and *RL*_*EW-K*_. Figures 5, 6 and 7 show similar results but just for different *G*_*tn*_ and *G*_*tt*_ sets. As shown by the Figs. 4, 5, 6, and 7, *RL*_*OPT-K*_ trained on only 5394 golden standard edges still performs better than the control methods that employ significantly more golden standard edges.

### Detection of interacting pairs far apart in the network

It is known that the basic idea of using random walk or random walk based kernels [17–20] for PPI prediction is that good interacting candidates usually are not faraway from the start node, e.g., only 2,3 edges away in the network. Consequently, for some existing network-level link prediction methods, testing nodes have been chosen to be within a certain distance range, which largely contributes to their good performance reported. In reality, however, a method that is capable and good at detecting interacting pairs far apart in the network can be even more useful, such as in uncovering cross talk between pathways that are not nearby in the PPI network.

**Fig. 8:**
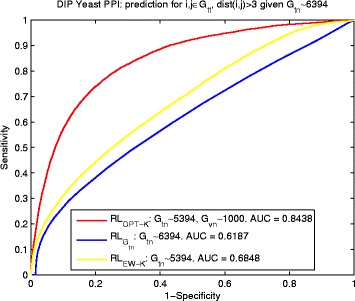
ROC curves of predicting $ G_{\textit {tt}}^{(dist(i,j)>3)} $ by $ RL_{G_{\textit {tn}}\sim 6394} $, *RL*_*OPT-K*_, and *RL*_*EW-K*_

To investigate how our proposed method performs at detecting faraway interactions, we still use $ RL_{G_{\textit {tn}}\sim 6394} $, *RL*_*OPT-K*_, and *RL*_*EW-K*_ for inferring PPIs, but we select node pairs (*i*,*j*) that satisfy *dist*(*i*,*j*)>3 *given* *G*_*tn*_∼6394 from *G*_*tt*_ as new testing set and name it $ G_{\textit {tt}}^{(dist(i,j)>3)} $. Figure 8 shows that *RL*_*OPT-K*_ has not only a significant margin over the control methods in detecting long-distance PPIs but also maintains a high ROC score of 0.8438 comparable to that of all PPIs. In contrast, $ RL_{G_{\textit {tn}}\sim 6394} $ performs poorly and worse than *RL*_*EW-K*_, which means the traditional RL kernel based on adjacent training network alone cannot detect faraway interactions well.

### Analysis of weights and efficiency

As the method incorporates multiple heterogeneous data, it can be insightful to inspect the final optimal weights. In our case, the optimal weights are 0.8608, 0.1769, 0.9334, 0, 0.0311, 0.9837, respectively for feature kernels *G*_*tn*_, *K*_*Jaccard*_, *K*_*SN*_, *K*_*B*_, *K*_*E*_, and *K*_*Pfam*_. These weights indicate that *K*_*SN*_ and *K*_*Pfam*_ are the predominant contributors to PPI prediction. This observation is consistent with the intuition that proteins interact via interfaces made of conserved domains [39], and PPI interactions can be classified based on their domain families and domains from the same family tend to interact [40–42]. Although the true strength of our method lies in integrating multiple heterogeneous data for PPI network inference, the optimal weights can serve as a guidance to select most relevant features when time and resources are limited.

Lastly, despite of the common concern of time efficiency with methods based on evolutionary computing, the issue is mitigated in our case. In our experiments, only a small number of particles, 150 to be exact, is needed for the initial population for ABC-DEP sampling. Also, as shown in the Fig. 2, our ABC-DEP algorithm is quickly converged, within 10 iterations. Moreover, since the PPI inference from *RL*_*OPT-K*_ is shown to be less sensitive to the size of training data, only 5394 gold standard edges, less than 25 % of the total number, are used. And, we do not need to retrain the model for different testing data, which is another time-saving property of our method.

## Conclusions

In this work, we developed a novel supervised method that enables inference of PPI networks from topological and genomic feature kernels in an optimized integrative way. Tested on DIP yeast PPI network, the results show that our method exhibits competitive advantages over control methods in several ways. First, the proposed method achieved superior performance in PPI prediction, as measured by ROC score, over 20 % higher than the baseline, and this margin is maintained even when the control methods use a significantly larger training set. Second, we also demonstrated that by integrating topological and genomic features into regularized Laplacian kernel, the method avoids the short-range problem encountered by random-walk based methods—namely the inference becomes less reliable for nodes that are far from the start node of the random walk, and show obvious improvements on predicting faraway interactions. Lastly, our method can also provide insights into the relations between PPIs and various similarity features of protein pairs, thereby helping us make good use of these features. As more features with respect to proteins are collected from various -omics studies, they can be used to characterize protein pairs in terms of feature kernels from different perspectives. Thus, we believe that our method provides a useful framework in fusing various feature kernels from heterogeneous data to improve PPI prediction.
